# Automatic Ergonomic Risk Assessment Using a Variational Deep Network Architecture

**DOI:** 10.3390/s22166051

**Published:** 2022-08-12

**Authors:** Theocharis Chatzis, Dimitrios Konstantinidis, Kosmas Dimitropoulos

**Affiliations:** Visual Computing Lab, Information Technologies Institute, Centre for Research and Technology Hellas, VCL of CERTH/ITI Hellas, 57001 Thessaloniki, Greece

**Keywords:** computer vision, deep learning, ergonomic risk assessment, work-related musculoskeletal disorders

## Abstract

Ergonomic risk assessment is vital for identifying work-related human postures that can be detrimental to the health of a worker. Traditionally, ergonomic risks are reported by human experts through time-consuming and error-prone procedures; however, automatic algorithmic methods have recently started to emerge. To further facilitate the automatic ergonomic risk assessment, this paper proposes a novel variational deep learning architecture to estimate the ergonomic risk of any work-related task by utilizing the Rapid Entire Body Assessment (REBA) framework. The proposed method relies on the processing of RGB images and the extraction of 3D skeletal information that is then fed to a novel deep network for accurate and robust estimation of REBA scores for both individual body parts and the entire body. Through a variational approach, the proposed method processes the skeletal information to construct a descriptive skeletal latent space that can accurately model human postures. Moreover, the proposed method distills knowledge from ground truth ergonomic risk scores and leverages it to further enhance the discrimination ability of the skeletal latent space, leading to improved accuracy. Experiments on two well-known datasets (i.e., University of Washington Indoor Object Manipulation (UW-IOM) and Technische Universität München (TUM) Kitchen) validate the ability of the proposed method to achieve accurate results, overcoming current state-of-the-art methods.

## 1. Introduction

Work-related musculoskeletal disorders (WMSDs) have been ranked among the most reported jobsite injuries affecting muscles, nerves, tendons, and joints [1]. WMSDs have been associated with several occupational risk factors, such as excessive force, awkward work postures, and prolonged sitting and standing [2,3]. Consequently, detecting the above factors is of vital significance in order to alleviate the painful and long-term effects of WMSDs.

To prevent WMSDs, it is essential to quantify exposure risk levels and develop tools to reduce the load to reasonable standards for workers [4]. To this end, observational methods, such as Rapid Upper Limb Assessment (RULA) [5], Rapid Entire Body Assessment (REBA) [6], and Ergonomic Assessment Worksheet (EAWS) [7], have recently emerged for use by ergonomists who are abandoning traditional direct measurement approaches [8]. This is due to the fact that observational techniques are inexpensive, convenient, and do not meddle with workers’ tasks [9]. RULA provides an ergonomic risk assessment of the upper body after considering the location of the neck, trunk, and upper limbs, along with the external loads applied to the body [5], while REBA extends RULA by taking into account the lower body (i.e., position of legs) as well [10]. EAWS is used to identify postures of the entire body, as well as their duration, and assigns risk scores to them.

In the literature, there are distinct categories of ergonomic risk assessment methodologies with different levels of precision [11]. Traditionally, ergonomic risk assessment is carried either by workers’ self-reports or by experts that observe and evaluate postures. However, such methods are time-consuming, cannot be applied in real-time, and are prone to errors [12]. In recent years, great advancements have been made on automatic ergonomic risk assessment [13], mainly due to the availability of wearable sensors [14,15] and the efficiency of deep learning models [16,17,18]. Nevertheless, the ideal sensors should fulfill certain unique characteristics, such as being easy to wear, unobtrusive, affordable, and wireless [19]. Furthermore, there is limited research work on methods that deal with ergonomic risk assessment in real-time or requiring only a single RGB image. Both of these factors are of crucial importance in order to substantially assist workers, preventing or relieving of WMSD symptoms and effects [20].

In this work, we introduce a novel deep learning-based method in order to decisively address the aforementioned challenging factors. We design a robust and generic framework that can regress total, as well as partial, REBA scores, given a single RGB image. The proposed approach extracts 3D pose information from images, computes a descriptive skeletal latent space through a multi-stream encoder and a multi-layer Transformer encoder, aligns the skeletal latent space with the ground truth REBA scores to improve the discrimination ability of the network, and provides accurate ergonomic risk assessment results in-real-time. The main contributions of this paper are:

- We propose a novel deep learning methodology that processes RGB images in real-time to assess ergonomic risk scores for the entire body and individual body parts unobtrusively, identifying which body parts are affected the most during a task;
- We introduce a novel variational framework that can effectively model and combine joint interactions with ergonomic risk information through the alignment of the 3D skeletal pose and the ground truth REBA scores, leading to the accurate estimation of ergonomic risks;
- We conduct thorough experiments on two well-known publicly available RGB datasets, UW-IOM [16], and TUM Kitchen [21], showcasing the superiority of the proposed methodology against other state-of-the-art methods.

## 2. Related Work

Methods that assess the ergonomic risk of work-related tasks can be classified into: (i) Traditional, (ii) Marker-based, and (iii) Marker-less ones. Works that fall under the first category rely upon either manual on-site observations or recorded videos and they are conducted by experts. Thus, these methods are time-consuming, may lead to subjective results due to observer bias and weariness and more importantly they cannot be employed in real-time.

On the other hand, Marker-based methods employ wearable equipment to acquire accurate information regarding human posture. Yan et al. [22] introduced a real-time motion warning personal protective equipment (PPE), using wearable Inertial Measurement Units (WIMUs) in order to detect predefined hazardous ergonomic postures and warn workers. Malaise et al. [23] utilized a wearable motion tracking suit and a sensorized glove to automatically recognize and classify different activities using a Hidden Markov Model. The same authors extended their previous work [24] by introducing a taxonomy of postures and actions, as well as proposing a system with a motion suit for automatic ergonomic risk assessment based on activity recognition, performed by a Hidden Markov Model. Mudiyanselage et al. [19] employed the surface electromyogram (sEMG) in order to automatically detect harmful lifting activities. Afterwards, the authors used the sEMG data to train four machine learning algorithms (i.e., SVM, KNN, Decision Tree, and Random Forest) to classify the level of ergonomic risk. Due to the inherent dynamic nature of work-related activities, not all sensors can be utilized for personalized safety monitoring [25]. Furthermore, signal artifacts and noise in wearable-sensors’ field measurements can be a challenging factor [26]. Consequently, such methods require expensive, cumbersome, and dedicated equipment, while also being sensitive to the surrounding environment and obtrusive to the actions of a worker.

Marker-less methods leverage deep learning techniques and most often incorporate the task of ergonomic risk assessment while performing action recognition. Abobakr et al. [27] introduced a deep learning-based framework in order to regress body joint angles from a single depth image, utilizing a deep residual network. Parsa et al. [16] utilized a CNN to learn spatial features from an input video that were then fed into a temporal CNN for real-time segmentation into meaningful actions. The same authors employed a pose extraction network to compute sequences of skeletons, which were then processed by a Spatio-Temporal Pyramid Graph Convolutional Network in order to perform action recognition and ergonomic risk assessment [28]. In a latter work, Parsa et al. [29] utilized a graph CNN alongside a temporal encoder–decoder for activity segmentation as well as an LSTM to simultaneously predict the total REBA score.

In a similar fashion, Li et al. [30] employed a deep learning-based method to extract 2D skeleton information from an RGB image. Afterwards, they used a regression network to predict the corresponding 3D pose and compute the total RULA score. The same authors [31] extended their previous work omitting the intermediate 3D skeletal representation, since it is more computationally expensive. Moreover, they fed the predicted 2D pose into a RULA estimator to predict RULA action levels instead of total scores, as the former ones are less susceptible to slight variations in rotation. Li et al. [32] performed motion analysis using images captured from two surveillance cameras. First, they extracted 2D joint coordinates and afterwards they reconstructed the corresponding 3D pose. Plantard et al. [33] used occlusion-resistant Kinect skeleton data correction to accurately compute joint angles and RULA scores. Mehrizi et al. [34] proposed a multi-view based deep perceptron approach. The authors extracted 2D shape and texture information from different views and then they employed a second module in order to predict 3D pose by synthesizing information from all available views. Konstantinidis et al. [17] extracted 3D skeletal poses from RGB images and then they regressed REBA scores using a multi-stream deep network that processed individually and then fused the pose information from different body parts.

On the other hand, modeling structured information, e.g., skeletal data, has become increasingly popular for various tasks, such as Human Action Recognition. The authors in [35] proposed a context aware graph convolutional network for the task of action recognition. The network considered a context term for each vertex by integrating information from all other vertices, thus modeling long range dependencies and removing the need of stacking multiple layers to enlarge the receptive field. Shi et al. [36] combined a multi-stream graph CNN that integrates the motion modality for both joints and the bones, with a spatial-temporal-channel (STC) attention module in order to perform skeleton-based action recognition. Plizzari et al. [37] proposed a Spatial-Temporal Transformer network to model dependencies between joints using the Transformer self-attention operator. The Spatial Self-Attention module captured intra-frame interactions between different body parts, while the Temporal Self-Attention module modelled inter-frame correlations.

Despite the satisfactory performance of the Marker-less methods, most of them are not capable of implementing real-time ergonomic risk assessment, since they usually perform action classification or segmentation in parallel, introducing additional computational burden. Furthermore, some of them either require explicitly a video as input or they are trained on specific actions, limiting their generalization capabilities. In this paper, we propose a novel deep learning-based framework that is able to perform ergonomic risk assessment irrespective of the work-related task performed. In addition, the proposed approach can regress total and partial REBA scores from a single RGB image, providing vital information to workers for potential harmful postures.

## 3. Methodology

**Figure 1:**
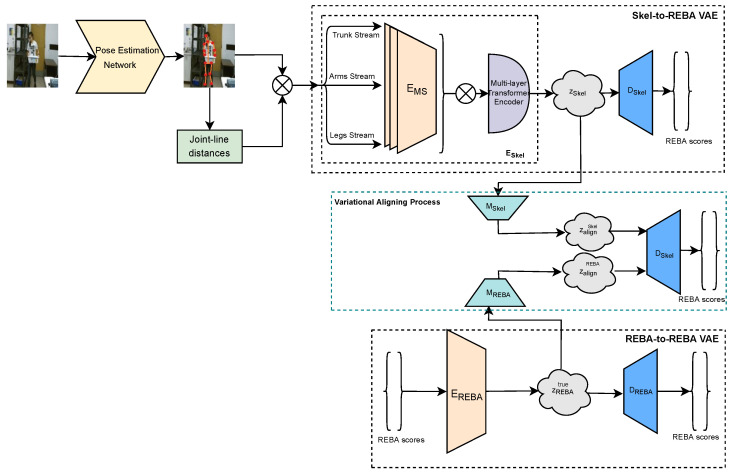
An overview of the proposed variational framework. Given an input image, 3D pose information is extracted from any human pose estimation algorithm and fed into a multi-stream encoder and a multi-layer Transformer encoder to model local and global joint interactions and generate the skeletal latent space. A second variational branch is employed to derive the true posterior distribution of REBA scores. Finally, the variational aligning process aims to bring the computed skeletal latent space closer to the one related to the ground truth REBA scores, enhancing the discrimination ability of the skeletal latent space and improving the ergonomic risk assessment results. The operator ⊗ denotes concatenation.

The aim of the proposed deep learning framework is to assess with high accuracy and robustness work-related ergonomic risks in the form of partial and total REBA scores through the processing of RGB images. The proposed framework constitutes a marker-less and cost-effective solution to the ergonomic risk assessment task, and it consists of the following three important modules with distinct functionalities. Firstly, the skeletal feature extraction and representation module are responsible for extracting 3D pose information from the RGB images. Subsequently, two variational networks, namely Skel-to-REBA and REBA-to-REBA, are employed to process the skeletal and ground truth REBA score information, respectively, derive discriminative latent spaces and accurately estimate partial and total REBA scores. Finally, the variational aligning process module is responsible for effectively aligning the two different latent spaces by bringing them close to each other so that the accuracy of the proposed ergonomic risk assessment framework is greatly improved. Figure 1 presents an overview of the proposed methodology. Next, the different modules of the proposed framework are described in detail.

### 3.1. Skeletal Feature Extraction and Representation

This module is responsible for the extraction of 3D skeletal information from a single RGB image, since skeletal information can limit the effect of irrelevant RGB context and improve the performance of the proposed ergonomic risk assessment method. To this end, we extract and employ 3D joint coordinates from a set of 14 widely used joints, as shown in Table 1, thus allowing any state-of-the-art human pose estimation network, such as VIBE [38] and METRO [39], to be successfully used for the joint coordinate computation.

Apart from the 3D joint coordinates, joint-line distances that measure the distances from each joint to the line shaped by the remaining joint pairs are also employed [40]. In the literature, several works [40,41,42,43] successfully adopted joint-line distances concluding that this alternative representation can better capture the relationship between joints and even require fewer training samples compared to raw joint coordinates. More specifically, the joint-line distance between the joint $J_{1}$ and the line formed by the joint pair $\left\{J_{2},J_{3}\right\}$, denoted by $L_{J_{2},J_{3}}$, is equal to the length of the perpendicular line between $J_{1}$ and $L_{J_{2},J_{3}}$ and its computation can be accelerated using Heron’s formula:

(1) $$S_{\Delta }\left(J_{1},L_{J_{2},J_{3}}\right)=2\frac{\sqrt{s\left(s-d_{J_{1},J_{2}}\right)\left(s-d_{J_{1},J_{3}}\right)\left(s-d_{J_{2},J_{3}}\right)}}{d_{J_{2},J_{3}}},$$

where $d_{J_{1},J_{2}}$, $d_{J_{1},J_{3}}$, $d_{J_{2},J_{3}}$ are the Euclidean distances between $J_{1},J_{2},J_{3}$ and $s=0.5(d_{J_{1},J_{2}}+d_{J_{1},J_{3}}+d_{J_{2},J_{3}})$.

We choose to utilize joint-line distances alongside 3D joint coordinates, since their combination is a more powerful and robust representation, leading to more accurate ergonomic risk assessment score predictions. Thus, we concatenate each 3D joint coordinate and the joint-line distances of this joint to the remaining ones to form a stream of 3D pose information for further processing.

### 3.2. Variational Encoders for Ergonomic Risk Assessment

The second module of our framework comprises two variational encoders (VAEs), namely Skel-to-REBA and REBA-to-REBA. The Skel-to-REBA VAE is responsible for processing joint information and modelling the interactions among joints to accurately predict ergonomic risk scores. At the core of its encoder, $E_{skel}$, lies a novel deep network architecture, consisting of a multi-stream joint encoder $E_{MS}$ and a multi-layer Transformer encoder $E_{T}$ that takes as input a group of joints, depicted in Table 2, and computes a highly descriptive latent space representation.

**Figure 2:**
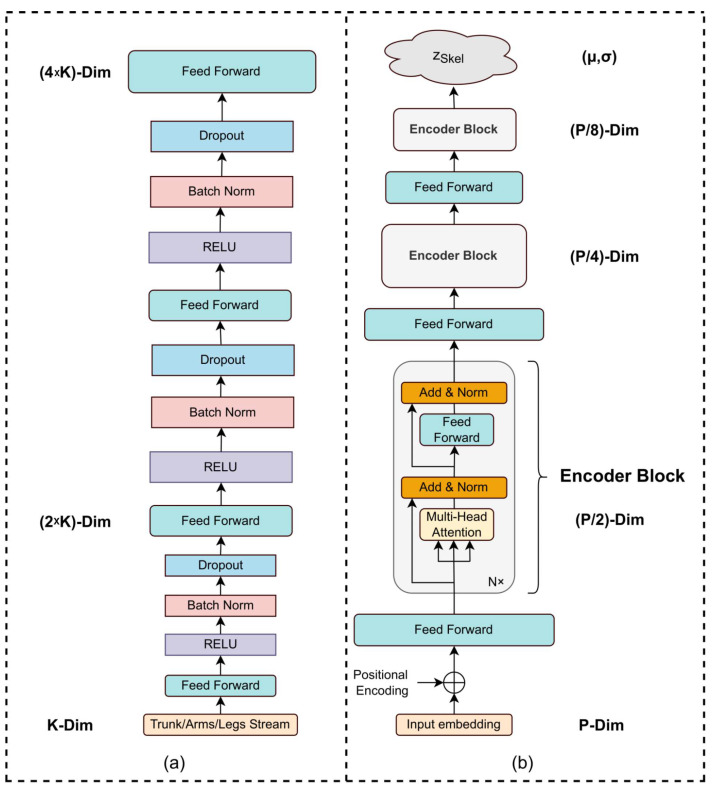
An overview of (**a**) the multi-stream encoder $E_{MS}$ and (**b**) the multi-layer Transformer encoder $E_{T}$. The multi-stream encoder $E_{MS}$ performs feature vector upsampling to each input stream of dimensionality K with the purpose to model the joint local relationships. The Transformer encoder $E_{T}$ performs self-attention through three encoder blocks and dimensionality reduction using linear projections. The final output is a pair of vectors (μ,σ) that composes the skeletal latent distribution. Each encoder block has N layers and H attention heads. P denotes the dimension of the concatenated input skeletal feature vector.

More specifically, the joint encoder $E_{MS}$ aims to model local spatial relationships among the different human body joints. The joint encoder $E_{MS}$ consists of a series of feedforward layers, as shown in Figure 2a, that process joint information split in three different data streams based on the location of the joints in the human body and inspired by the manual REBA computation procedure. Subsequently, the outputs from the different data streams of the joint encoder $E_{MS}$ are concatenated to form the input for the Transformer encoder $E_{T}$. The purpose of the Transformer encoder $E_{T}$ is to identify and model the global relationship among the human body joints to further enhance the discrimination ability of the computed skeletal latent space. To this end, the architecture of the proposed Transformer encoder (shown in Figure 2b) is inspired by [39] that uses feed-forward layers to reduce the dimensionality of the hidden embedding after each encoder layer. The output of the encoder $E_{Skel}$ is fixed-size vectors $\mu_{Skel}$ and $\sigma_{Skel}$ that constitute the skeletal latent distribution ($z_{skel}$), with dimensionality $d_{Skel}$, which parametrize a Gaussian distribution $N(\mu_{Skel},\Sigma_{Skel})$, where $\Sigma_{Skel}=diag\left(\sigma_{Skel}{\left(1\right)}^{2},\ldots ,\sigma_{Skel}{\left(d_{Skel}\right)}^{2}\right)$.

Afterwards, we stochastically draw a sample from the skeletal latent space, using the $D_{Skel}$ decoder, to regress the partial and total REBA scores, S=$\{s_{neck},s_{trunk},$$s_{lower\_arms},s_{upper\_arms},s_{total}\}$. Since our framework is variational, we need to incorporate the regularization scheme. Thus, we aim to use the Kullback–Leibler divergence to bring the skeletal latent distribution as close as possible to a standard multivariate normal distribution. The second objective aims to minimize the MSE loss between the ground truth (*y*) and the predicted ($\hat{y}$) REBA scores. Consequently the weights of the Skel-to-REBA VAE are optimized according to the following objective:

(2) $$\begin{array}{c} \begin{array}{cc} L_{VAE}^{Skel} & =\beta L_{KL}^{Skel}+L_{MSE}^{Skel}=\\ \; & =\beta \frac{1}{2}\sum_{j=1}^{d_{Skel}}\left(\sigma_{Skel}^{2}\left(j\right)+\mu_{Skel}^{2}\left(j\right)-\ln\sigma_{Skel}^{2}\left(j\right)-1\right)+\frac{1}{K}\sum_{j=1}^{K}{\left(y\left(j\right)-{\hat{y}}_{i}\left(j\right)\right)}^{2}, \end{array} \end{array}$$

where *K* is the dimensionality of the target/REBA score modality.

On the other hand, the REBA-to-REBA VAE aims to create a well-structured latent space that contains significant information related to ergonomic scores, by reconstructing the true posterior distribution. To achieve this, we employ the reconstruction encoder, $E_{REBA}$, to encode the ground truth scores $S\in R^{6}$ into $\left(\mu_{REBA}^{true},\sigma_{REBA}^{true}\right)$, thus generating the true posterior distribution of the REBA scores, $z_{REBA}^{true}$. Then, we similarly draw a sample from this latent space and decode it using the $D_{REBA}$ decoder, to infer the REBA scores. The objective of this branch can be modelled as:

(3) $$\begin{array}{c} \begin{array}{cc} L_{VAE}^{REBA} & =\beta L_{KL}^{REBA}+L_{MSE}^{REBA}=\\ \; & =\beta \frac{1}{2}\sum_{j=1}^{d_{REBA}}\left(\sigma_{REBA}^{2}\left(j\right)+\mu_{REBA}^{2}\left(j\right)-\ln\sigma_{REBA}^{2}\left(j\right)-1\right)+\frac{1}{K}\sum_{j=1}^{K}{\left(x\left(j\right)-{\hat{x}}_{i}\left(j\right)\right)}^{2}, \end{array} \end{array}$$

where *x* denotes the ground truth and $\hat{x}$ the predicted REBA scores from the REBA-to-REBA VAE.

### 3.3. Variational Aligning Process

The purpose of the proposed variational aligning process is to effectively bring closer the skeletal latent space $z_{skel}$ with the latent space of the ground truth REBA scores $z_{REBA}^{true}$. The reason behind this is that the REBA-to-REBA VAE is able to create a latent space representation that can more accurately model the ergonomic risk information. In this context, the REBA-to-REBA VAE is employed as a teacher network to guide the Skel-to-REBA VAE towards predicting more robust REBA scores. To achieve this, we employ two variational alignment components, $M_{Skel}$ and $M_{REBA}$, that aim to project the skeletal and ergonomic risk latent distributions into new ones that can be more easily aligned to each other. Subsequently, we use the pretrained Skel-to-REBA decoder, $D_{Skel}$, in order to alternatively decode samples drawn from the above latent spaces. The common decoding scheme of $D_{Skel}$ aims to bring closer these latent distributions through a training to correctly classify both of them, thus contributing to the creation of a more meaningful and informative skeletal latent space. More specifically, $M_{Skel}$ gets as input the vectors of mean and variance that are generated by $E_{Skel}$, ($\mu_{Skel},\sigma_{Skel})$ and outputs a new latent distribution, with mean $\mu_{align}^{Skel}$ and variance $\sigma_{align}^{Skel}$. In a similar way, $M_{REBA}$ takes the pair ($\mu_{REBA},\sigma_{REBA}$) and produces a new latent space that can be described as ($\mu_{align}^{REBA},\sigma_{align}^{REBA}$).

In order to train our network, we optimize the common VAE loss for both the new generated latent distributions and the MSE loss. Consequently, the objective can be formulated as:

(4) $$L_{align}=L_{align}^{Skel}+\gamma L_{align}^{REBA},$$

where γ denotes the weight of the REBA-to-REBA loss and:

(5) $$\begin{array}{c} \begin{array}{cc} L_{align}^{Skel} & =\beta L_{KL}^{Skel_{align}}+L_{MSE}^{Skel_{align}}=\\ \; & =\beta \frac{1}{2}\sum_{j=1}^{d_{Skel}}\left({\sigma_{align}^{Skel}}^{2}\left(j\right)+{\mu_{align}^{Skel}}^{2}\left(j\right)-\ln{\sigma_{align}^{Skel}}^{2}\left(j\right)-1\right)+\frac{1}{K}\sum_{j=1}^{K}{\left(y\left(j\right)-{\hat{y}}_{i}^{align}\left(j\right)\right)}^{2} \end{array} \end{array}$$

and

(6) $$\begin{array}{c} \begin{array}{cc} L_{align}^{REBA} & =\beta L_{KL}^{REBA_{align}}+L_{MSE}^{REBA_{align}}=\\ \; & =\beta \frac{1}{2}\sum_{j=1}^{d_{REBA}}\left({\sigma_{align}^{REBA}}^{2}\left(j\right)+{\mu_{align}^{REBA}}^{2}\left(j\right)-\ln{\sigma_{align}^{REBA}}^{2}\left(j\right)-1\right)+\frac{1}{K}\sum_{j=1}^{K}{\left(x\left(j\right)-{\hat{x}}_{i}^{align}\left(j\right)\right)}^{2}, \end{array} \end{array}$$

where ${\hat{y}}^{align},{\hat{x}}^{align}$ denote the predicted REBA scores regressed from the Skel-to-REBA and REBA-to-REBA VAE, correspondingly.

It should be noted that, during this process, a fine-tuning of the pre-trained encoders $E_{Skel}$ and $E_{REBA}$ is performed.

## 4. Experiments

### 4.1. Datasets and Metrics

The proposed method is tested on two publicly available datasets, namely University of Washington Indoor Object Manipulation (UW-IOM) and Technische Universität München (TUM) Kitchen.

The UW-IOM dataset contains videos of 20 individuals picking up and placing objects of varying weights from and to cabinets and tables located at various heights. This dataset consists of 17 action classes, each following a four-tier hierarchy denoting the object manipulation, human motion, type of object manipulation and the relative height of the surface on which the activity is taking place (low, medium, and high).

The TUM Kitchen dataset consists of 20 videos captured by four static monocular cameras with overlapping views. Each video depicts daily actions performed by an individual in a kitchen, involving walking, picking up, and placing objects from and to drawers, tables and cabinets. The average duration of the videos are about two minutes. The actions are manually labeled and provided separately for the left hand, the right hand, and the trunk of the person.

Following previous works, we employ a cross-validation approach by splitting both datasets into four subsets and using three subsets for training and 1 for evaluation, each time. Additionally, we utilize the provided annotations in order to temporally crop the videos. Finally, we report on the most common metrics, being the mean squared error (MSE), the root mean squared error (RMSE), and the mean absolute error (MAE), in order to compare the ground truth and the predicted partial and total REBA scores.

### 4.2. Implementation Details

In order to acquire the ground truth REBA scores for both datasets, we follow a similar scheme to previous works [17,29]. More specifically, the VIBE 3D pose estimation algorithm is employed to extract the 3D joint coordinates of the human body. The extraction of 3D pose information is essential for the accurate computation of joint angles due to the distortion the angles suffer from, when they are projected into the 2D image plane [44]. Next, the joint angles (e.g., flexion, abduction, etc.) among all body parts are computed, and the REBA framework with its proposed calculations is followed to compute partial and total ground truth REBA scores.

Since REBA scores are discrete integers from 1 to 15, the minimum and maximum risk level; correspondingly, the computed ground truth REBA scores are sequences of piece-wise constants. Nevertheless, training the REBA decoder on such sequences is difficult and thus we opt to smooth the REBA scores using a Savitzky–Golay filter with a kernel of size 12 for the UW-IOM and 25 for the Kitchen dataset, based on their average fps. Regarding the joint coordinates that VIBE estimates, we translate them to the neck before we feed them into the rest of the network, in order to make the skeletal features invariant to the absolute position of the subject in the image, thus enhancing the performance capabilities of the proposed method.

The first module of the Skel-to-REBA VAE, i.e., the multi-stream encoder $E_{MS}$, consists of four fully connected layers per stream as shown in Figure 2a and upsamples the input feature vectors, leading to an output vector of 2048 dimensionality. On the other hand, the Transformer encoder $E_{T}$ consists of three encoder blocks with four layers and four attention heads each, as shown in Figure 2b. The output of the Transformer encoder constitutes a pair of vectors (μ,σ) of 128 dimensionality. As far as REBA-to-REBA encoder and decoder, $E_{REBA}$ and $D_{REBA}$, and the Skel-to-REBA decoder, $D_{Skel}$, are concerned, we use six fully connected layers. The architecture of the VAE alignment components is similar to [45]. The dimensions of the latent space are set to 128 for the Skel-to-REBA VAE and 64 for the REBA-to-REBA VAE. We use the Adam optimizer [46] with learning rate $1_{}^{-4}$ and batch size of 128. We set the weight of the Kullback–Leibler divergence β to $1_{}^{-4}$ and the weight of the REBA-to-REBA VAE during the final Variational Aligning Process γ to $1_{}^{-2}$. All the aforementioned hyperparameters were chosen empirically since they provided the optimal results during our experiments.

The network training takes place in two phases. During the first phase, the two VAE branches are trained independently of each other. Subsequently, after they converge, we employ the variational aligning components in order to bring the skeletal information closer to the ground truth REBA information.

For the experiments, we use the PyTorch [47] Deep Learning framework and a PC with Intel 8700 K (4.7 GHz) CPU, Nvidia GTX 1080Ti (11GB VRAM) GPU, and 32 GB RAM. Finally, the most computationally expensive part of our method is the skeletal feature extraction. The VIBE algorithm has a runtime speed of 15–20 fps, while the joint-line distances computation and the REBA scores regression introduce a small additional computational burden. Thus, the proposed deep network architecture is able to achieve a processing speed of 9–14 fps, making it suitable for real-time applications.

### 4.3. Experimental Results

We compare the performance of our proposed method against [17,29], since these are the only methods that carry out the task of ergonomic risk assessment in real-time, using the REBA framework. Parsa et al. [29] perform action segmentation and incorporate the predicted action, by fusing activity embedding with spatial features (MTL-emb), in order to predict more accurate REBA scores. Thus, they present two results based on whether or not they use these embeddings. Although the results in which the action embedding is incorporated produce more accurate REBA scores, the authors used a supervised learning framework that constrained the usability of their method only to the trained activities. Moreover, it should be noted that this work requires a video sequence as input, while our approach is capable of regressing REBA scores given either a single RGB image or a video sequence. On the other hand, Konstantinidis et al., in their work [17] that we name MSDN for short notation, is able to regress both partial and total REBA scores using a multi-stream deep network architecture and intermediate guidance for partial score regression. The proposed work takes as input the same skeletal information and produces the same type of output as the MSDN method. However, the two methods differ in the deep network architecture that is used to process the skeletal information and predict the REBA scores, as well as the fact that the proposed method employs the ground truth ergonomic risk scores as input through a variational framework to further improve the ergonomic risk assessment results.

Table 3 summarizes the performance of our proposed methodology against the above state-of-the-art approaches. Our proposed variational framework outperforms all state-of-the-art methods, yielding 0.297 and 0.265 MSE in the UW-IOM and TUM Kitchen datasets, respectively. Regarding [29], it can be deduced that our method produces considerably more accurate ergonomic risk scores despite the fact that we do not enhance the network performance by utilizing action classes. Consequently, our framework can be used as a generalized risk assessment framework for any work-related task. This deduction is further amplified by the flexible input requirements of the proposed methodology (i.e., processing of images irrespective of the task) combined with the effectiveness shown in the two datasets, in which the workers perform highly different tasks (i.e., moving and placing heavy objects in cabinets in UW-IOM against moving and placing plates and kitchen utensils in a kitchen environment in TUM-Kitchen). As far as [17] is concerned, the proposed methodology provides a relative decrease in MSE of 4% and 5% in the UW-IOM and the TUM Kitchen datasets, respectively. In addition, Table 4 and Table 5 present a comparison of partial and total ergonomic risk scores in terms of MSE, MAE, and RMSE on both datasets. It can be noted that our framework produces more accurate predictions for each individual body part as well as the entire body, compared to the method of [17], verifying the importance of utilizing a variational approach for improved generalization and employing the ground truth REBA scores to guide the network towards improved ergonomic risk assessment performance. In addition, the accurate estimation of partial REBA scores can provide valuable feedback regarding which body parts receive the most strain during a work-related task.

Table 6 displays the distribution of the ground truth and predicted REBA risk levels for both datasets. The REBA framework defines the following risk levels: Negligible (REBA score <2), Low (2≤ REBA score <4), Medium (4≤ REBA score <8), High (8≤ REBA score <11) and Very High (REBA score ≥11). As we can see, the percentage of the frames predicted as at a Very High risk level is very close to the corresponding ground truth ones, regardless of the fact that there is a limited number of such instances on both datasets (i.e., 0.02% and 0.07%, in the UW-IOM and TUM Kitchen datasets, respectively). On the other hand, a few discrepancies with regard to ground truth risk levels can be detected on postures that fall under Low risk levels category, which are assessed as Medium ones, probably due to the fact that both datasets consists of imbalanced risk level classes. In addition, it can be noticed that the predicted risk scores for certain body parts, such as neck and lower arms, are very close to ground truth scores, while the estimated total REBA scores present the largest deviations.

**Figure 3:**
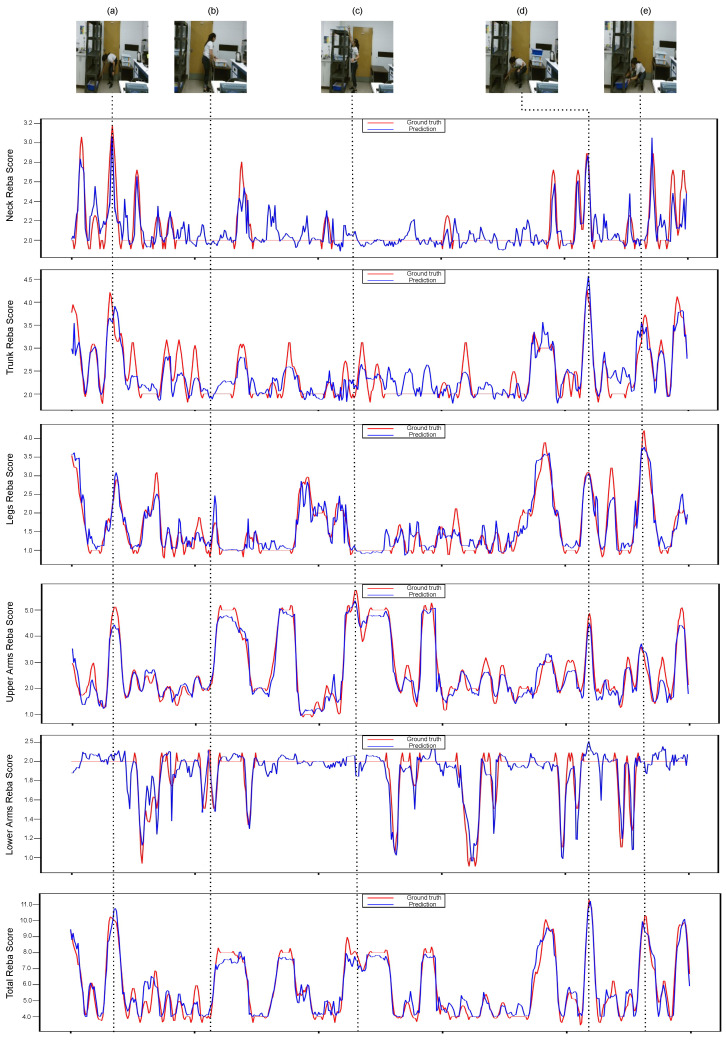
Visualization of ground truth (red lines) and predicted (blue lines) partial and total REBA scores on a video sequence from the UW-IOM dataset. At the top, five frames (**a**–**e**) that correspond to extreme postures with high ergonomic risks are displayed, while REBA scores for individual body parts and the whole body follows.

Furthermore, Figure 3 demonstrates the ground truth and the predicted REBA scores for each individual body part, as well as the whole body, for a video sample from the UW-IOM dataset. A visualization of the results verify the effectiveness of the proposed method to accurately predict both partial and total REBA scores for different postures. For instance, in Figure 3c, the worker is in an upright position with an extended arm and the predicted REBA scores for the neck and legs are low (<2), while for the upper arms is high (>5). On the other hand, in Figure 3e, the worker is in a sitting position leaning forward and putting significant pressure on her trunk and legs, increasing the predicted REBA scores for these body parts to over 3.5, while the predicted REBA scores for the remaining body parts are lower. Similar conclusions can be drawn for other postures as well.

From the experimental results, we can observe that the proposed methodology surpasses all state-of-the-art methods using the MSE metric in the two tested datasets, while it outperforms the MSDN method in terms of both partial and total ergonomic risk scores using MSE, MAE, and RMSE. Regarding the computational cost, it should be noted that the proposed network is slightly slower than the MSDN one, since it consists of about 30% more parameters; however, it can achieve comparable processing speed of 9–14 fps against the corresponding performance of MSDN, which is 10–15 fps. These comparisons demonstrate the effectiveness of combining local and global joint modelling, as well as taking into consideration the ground truth REBA scores to create a more discriminative skeletal latent space. More importantly, we can observe that, in the more challenging TUM Kitchen dataset that includes occlusions due to the camera position, our method significantly improves on previous works, proving its high generalization capabilities.

## 5. Conclusions

This paper introduces a novel generic approach for automatic ergonomic risk assessment without resorting to obtrusive wearable sensors and irrespective of the nature of the work task. The proposed method provides ergonomic risks scores, according to the REBA framework, for different body parts and the entire body with high accuracy and robustness using 3D skeletal information (i.e., joint coordinates and joint-line distances) extracted from RGB images. Through a variational approach, the proposed method processes the skeletal information to accurately model the local and global interactions among different joints and form a descriptive skeletal latent space that can robustly represent human postures. In addition, a second network stream processes the ground truth ergonomic risk scores to extract important knowledge that is then embedded in the skeletal latent space in order to improve its discrimination ability and guide the network towards improved results. Experimental results in two challenging datasets, namely UW-IOM and TUM Kitchen, demonstrate the ability of the proposed method to achieve high accuracy and robustness (MSE < 0.3), overcoming the performance of state-of-the-art approaches. Additionally, the proposed method can predict very accurately ergonomic risk scores for different body parts, providing important feedback to workers regarding which body parts receive the most strain during a work-related task. Finally, the results on the classification of human actions in REBA risk levels show that the proposed method can successfully classify actions and, especially, identify very high risk actions despite being a small portion of the total number of actions in the datasets. 

## Figures and Tables

**Table 1:** The selected joints employed in the proposed methodology.

Selected Joints	
Head	Left Wrist
Neck	Right Hip
Right Shoulder	Left Hip
Left Shoulder	Right Knee
Right Elbow	Right Elbow
Left Elbow	Right Ankle
Right Wrist	Left Ankle

**Table 2:** The proposed network employs three streams that contain: trunk joints, arms joints, and legs joints.

Trunk Stream	Arms Stream	Legs Stream
Head	Head	Head
Neck	Neck	Neck
Right Shoulder	Right Hip	Right Shoulder
Left Shoulder	Left Hip	Left Shoulder
Right Hip	Right Knee	Right Elbow
Left Hip	Left Knee	Right Elbow
	Right Ankle	Right Wrist
	Left Ankle	Right Wrist

**Table 3:** Comparison against state-of-the-art approaches in the UW-IOM and TUM Kitchen datasets.

Method	UW-IOM	TUM Kitchen
MTL-base	0.89 ± 0.24	1.18 ± 0.68
MTL-emb	0.61 ± 0.36	1.11 ± 0.38
MSDN	0.31 ± 0.04	0.28 ± 0.03
Proposed	0.297 ± 0.03	0.265 ± 0.04

**Table 4:** Performance of the proposed method in terms of partial and total REBA scores in the UW-IOM dataset.

REBA Scores	UW-IOM					
	Proposed			MSDN		
	MSE	MAE	RMSE	MSE	MAE	RMSE
Neck	0.024 ± 0.002	0.113 ± 0.02	0.168 ± 0.04	0.03 ± 0.003	0.117 ± 0.03	0.171 ± 0.03
Trunk	0.075 ± 0.061	0.211 ± 0.03	0.278 ± 0.03	0.079 ± 0.065	0.217 ± 0.02	0.281 ± 0.04
Legs	0.071 ± 0.082	0.186 ± 0.03	0.261 ± 0.05	0.075 ± 0.077	0.192 ± 0.04	0.265 ± 0.05
Upper arms	0.095 ± 0.029	0.221 ± 0.04	0.301 ± 0.04	0.098 ± 0.023	0.226 ± 0.05	0.306 ± 0.03
Lower arms	0.015 ± 0.002	0.076 ± 0.03	0.119 ± 0.03	0.017 ± 0.001	0.079 ± 0.03	0.121 ± 0.04
Total	0.297 ± 0.032	0.377 ± 0.04	0.531 ± 0.06	0.31 ± 0.04	0.395 ± 0.05	0.557 ± 0.07

**Table 5:** Performance of the proposed method in terms of partial and total REBA scores in the TUM Kitchen dataset.

REBA Scores	TUM-Kitchen					
	Proposed			MSDN		
	MSE	MAE	RMSE	MSE	MAE	RMSE
Neck	0.018 ± 0.002	0.090 ± 0.02	0.135 ± 0.04	0.02 ± 0.003	0.093 ± 0.02	0.139 ± 0.03
Trunk	0.053 ± 0.009	0.159 ± 0.03	0.228 ± 0.03	0.055 ± 0.008	0.161 ± 0.03	0.236 ± 0.04
Legs	0.085 ± 0.005	0.213 ± 0.04	0.276 ± 0.05	0.088 ± 0.002	0.221 ± 0.05	0.286 ± 0.04
Upper arms	0.061 ± 0.018	0.186 ± 0.03	0.242 ± 0.03	0.065 ± 0.016	0.194 ± 0.04	0.251 ± 0.03
Lower arms	0.012 ± 0.002	0.067 ± 0.02	0.109 ± 0.02	0.013 ± 0.002	0.069 ± 0.02	0.114 ± 0.02
Total	0.265 ± 0.042	0.364 ± 0.04	0.499 ± 0.05	0.28 ± 0.03	0.389 ± 0.05	0.529 ± 0.06

**Table 6:** Ground truth and predicted REBA risk level distribution on the UW-IOM and TUM Kitchen datasets.

REBA Risk Level	UW-IOM		TUM Kitchen	
	Ground Truth	Predicted	Ground Truth	Predicted
Negligible	0%	0%	0%	0%
Low	12.32%	3.84%	12.07%	6.24%
Medium	79.34%	89.23%	76.44%	83.79%
High	8.32%	6.91%	11.42%	9.89%
Very High	0.02%	0.02%	0.07%	0.08%
